# Genome-wide association study meta-analysis identifies five new loci for systemic lupus erythematosus

**DOI:** 10.1186/s13075-018-1604-1

**Published:** 2018-05-30

**Authors:** Antonio Julià, Francisco Javier López-Longo, José J. Pérez Venegas, Silvia Bonàs-Guarch, Àlex Olivé, José Luís Andreu, Mª. Ángeles Aguirre-Zamorano, Paloma Vela, Joan M. Nolla, José Luís Marenco de la Fuente, Antonio Zea, José María Pego-Reigosa, Mercedes Freire, Elvira Díez, Esther Rodríguez-Almaraz, Patricia Carreira, Ricardo Blanco, Víctor Martínez Taboada, María López-Lasanta, Mireia López Corbeto, Josep M. Mercader, David Torrents, Devin Absher, Sara Marsal, Antonio Fernández-Nebro

**Affiliations:** 1 Rheumatology Research Group, Vall d’Hebron Research Institute, 08035 Barcelona, Spain; 2 Department of Rheumatology, Hospital Universitario Gregorio Marañón, 28007 Madrid, Spain; 3 Department of Rheumatology, Hospital del SAS de Jerez de la Frontera, 11407 Cádiz, Spain; 4 Barcelona Supercomputing Center. Joint BSC-CRG-IRB Research Program in Computational Biology, 08034 Barcelona, Spain; 5 Department of Rheumatology, Hospital Universitari Germans Trias i Pujol, 08916 Badalona, Spain; 6 Department of Rheumatology, Hospital Universitario Puerta de Hierro, 28222 Madrid, Spain; 7 Department of Rheumatology, Hospital Universitario Reina Sofía, 14004 Córdoba, Spain; 8 Department of Rheumatology, Hospital General Universitario de Alicante, 03010 Alicante, Spain; 9 Department of Rheumatology, Hospital Universitari de Bellvitge, 08907 Barcelona, Spain; 10 Department of Rheumatology, Hospital de Valme, 41007 Sevilla, Spain; 11 Department of Rheumatology, Hospital Universitario Ramón y Cajal, 28034 Madrid, Spain; 12 Department of Rheumatology, Hospital do Meixoeiro, Grupo IRIDIS, Instituto de Investigación sanitaria Galicia Sur (IISGS), 36312 Vigo, Spain; 13 Department of Rheumatology, Hospital Universitario A Coruña, 15006 A Coruña, Spain; 14 Department of Rheumatology, Hospital Complejo Asistencial Universitario de León, 24071 León, Spain; 15 Department of Rheumatology, Hospital Universitario 12 de Octubre, 28041 Madrid, Spain; 16 Department of Rheumatology, Hospital Universitario Marqués de Valdecilla, 39008 Santander, Spain; 17 Institució Catalana de Recerca i Estudis Avançats (ICREA), 08010 Barcelona, Spain; 18 HudsonAlpha Institute for Biotechnology, Huntsville, AL 35806 USA; 19 Department of Rheumatology, Hospital Regional Universitario de Málaga, Instituto de Investigación Biomédica de Málaga, 29011 Málaga, Spain

**Keywords:** Systemic lupus erythematosus, Genetic susceptibility, Genome-wide association study, Meta-analysis, Biological pathway analysis

## Abstract

**Background:**

Systemic lupus erythematosus (SLE) is a common systemic autoimmune disease with a complex genetic inheritance. Genome-wide association studies (GWAS) have significantly increased the number of significant loci associated with SLE risk. To date, however, established loci account for less than 30% of the disease heritability and additional risk variants have yet to be identified. Here we performed a GWAS followed by a meta-analysis to identify new genome-wide significant loci for SLE.

**Methods:**

We genotyped a cohort of 907 patients with SLE (cases) and 1524 healthy controls from Spain and performed imputation using the 1000 Genomes reference data. We tested for association using logistic regression with correction for the principal components of variation. Meta-analysis of the association results was subsequently performed on 7,110,321 variants using genetic data from a large cohort of 4036 patients with SLE and 6959 controls of Northern European ancestry. Genetic association was also tested at the pathway level after removing the effect of known risk loci using PASCAL software.

**Results:**

We identified five new loci associated with SLE at the genome-wide level of significance (*p* < 5 × 10^− 8^): *GRB2*, *SMYD3*, *ST8SIA4*, *LAT2* and *ARHGAP27*. Pathway analysis revealed several biological processes significantly associated with SLE risk: B cell receptor signaling (*p* = 5.28 × 10^− 6^), CTLA4 co-stimulation during T cell activation (*p* = 3.06 × 10^− 5^), interleukin-4 signaling (*p* = 3.97 × 10^− 5^) and cell surface interactions at the vascular wall (*p* = 4.63 × 10^− 5^).

**Conclusions:**

Our results identify five novel loci for SLE susceptibility, and biologic pathways associated via multiple low-effect-size loci.

**Electronic supplementary material:**

The online version of this article (10.1186/s13075-018-1604-1) contains supplementary material, which is available to authorized users.

## Background

Systemic lupus erythematosus (SLE [MIM: 152700]) is a common systemic autoimmune disease characterized by the production of autoantibodies and a complex genetic inheritance. The prevalence of the disease varies according to the population ancestry, with European populations ranging between 30 and 90 cases per 100,000 individuals [1]. SLE afflicts women at a rate nine times higher than men, and most often appears during childbearing ages. Concordance rate studies in monozygotic and dizygotic twins and recurrence risk estimates in siblings of probands (λ_s_), have clearly shown the importance of genetic factors in the development of the disease [2].

Despite the evidence for a strong genetic contribution, until recently, very few loci were convincingly associated with SLE risk [3]. With the concurrent identification of common genome variation and the development of genome-wide genotyping technologies, genome-wide association studies (GWAS) have dramatically changed the ability to identify risk variants. In SLE, GWAS have allowed the identification of more than 50 risk loci at a genome-wide significance level (*p* value <5 × 10^− 8^) [4–6]. These findings are of great relevance since they pinpoint specific biological mechanisms that are relevant for the disease and that otherwise would not have been prioritized for research [7]. In a severe disease like SLE that is lacking efficacious treatments, genetic studies provide a unique way to expand the number of molecular targets for drug discovery [8].

To date, the explanation for the inherited risk of SLE is largely unresolved. Including all known risk variants, less than 30% of disease heritability is currently accounted for [9], In order to identify additional risk variants, GWAS meta-analyses from different countries have proven to be a highly successful approach [9]. Currently, most Southern European populations have been underrepresented in GWAS of SLE. In Spain, epidemiological studies have shown that there is an increased prevalence of the disease compared to other European regions [1]. Consequently, the analysis of the genetic variation in this population could be highly useful to identify new genetic variation for SLE risk.

Biological pathways integrate the function of multiple genes and, therefore, provide a higher level of detection of the relevant genetic risk [10, 11]. To date, different statistical methods have been developed that exploit the biological knowledge in order to leverage the power of GWAS. These analysis methods are designed to aggregate the genetic evidence from multiple risk loci into a single association statistic. The use of cumulative evidence can be a powerful way to detect genetic associations and biological mechanisms that otherwise would have been missed due to low effect size at the single-marker level. Using this complementary GWAS approach, relevant biological insights have been gained in different complex diseases, including autoimmune diseases [12].

The aim of the current work was to identify new genetic risk loci for SLE by a GWAS meta-analysis using a case-control cohort from a previously untargeted population. After excluding known risk genes, pathway meta-analysis was also performed to identify biologic pathways for SLE risk associated by risk loci as yet unaccounted for.

## Methods

### Study cohorts

Patients and controls from the Spanish population were recruited through the Immune-Mediated Inflammatory Disease (IMID) Consortium [13]. Patients with SLE were recruited via the rheumatology departments of 17 university hospitals in Spain. All included patients fulfilled the 1982 revised American College for Rheumatology diagnosis criteria for SLE [14]. All patients were > 16 years old at the time of recruitment, although disease could have started earlier. A minimal disease evolution period of 3 years since diagnosis was also required for inclusion in this study. All Patients with SLE were Caucasian with all four grandparents born in Spain. Patients with an additional rheumatologic disease (e.g. rheumatoid arthritis, systemic sclerosis or mixed connective tissue disease) except antiphospholipid syndrome or Sjögren’s syndrome were excluded from the study. Also, patients with concomitant psoriasis or inflammatory disease (Crohn’s disease or ulcerative colitis) were also excluded from the study. A total of 907 patients with SLE were recruited for the GWAS. Additional file 1: Table S1 summarizes the main features of the Spanish GWAS cohort.

Healthy control individuals were also recruited through the IMID Consortium as described previously [15]. All controls were Caucasian and > 18 years old at the time of recruitment. Individuals with one or more grandparents born outside of Spain were excluded. Controls with an autoimmune disease or with a family history of autoimmune disease were also excluded from this cohort. A total of 1524 healthy control individuals were finally included in the present GWAS. All the procedures were followed in compliance with the principles of the Declaration of Helsinki and informed consent was obtained from all participants. The study and the consent procedure were reviewed and approved by the local institutional review boards.

GWAS data from European-ancestry cohorts were obtained from a recent meta-analysis [4]. For the present study, GWAS association data were obtained from 4036 patients with SLE and 6959 controls of Caucasian European ancestry. The details on the data quality control, imputation and statistical association analyses have been previously described [4]. Association data on a total of 37,577,690 markers from the 22 autosomal chromosomes were available for meta-analysis.

### Genotyping, quality control and imputation

In the Spain cohort, genome-wide genotyping was performed using the Illumina Quad610 Beadchips (Illumina, San Diego, CA, USA) at the National Genotyping Center (CeGen, Madrid, Spain). This array genotyping platform includes information on > 550,000 single nucleotide polymorphisms (SNPs). A whole blood sample (5 mL) was collected from all patients and genomic DNA extracted using the Chemagic Magnetic Separation Module I (PerkinElmer, Waltham, MA, USA). Genotyping was performed following the protocol recommended by Illumina. Genotype calling was performed using the GenomeStudio data analysis software v2011.1 (Illumina, San Diego, CA, USA). Genotyping quality control was performed using PLINK genomic analysis software [16]. Principal components of variation were estimated using Eigensoft (v4.2) software [17]. The genomic inflation factor was λ_GC_ = 1.16 in the European-ancestry GWAS, and λ_GC_ = 1.06 in the Spain GWAS (Additional file 1: Figure S2). After quality control analysis, 864 patients with SLE and 1513 controls were available for imputation.

Genome-wide imputation was performed using GUIDANCE, an integrated framework for haplotype phasing and genotype imputation of genotypes [18]. Markers and samples were first tested for quality control. SNPs with a genotyping call rate < 95% or a significant deviation from Hardy-Weinberg equilibrium in controls (*p* value ≤1 × 10^− 6^) were removed. Individuals with a genotype call rate < 95% or outlier genetic background (i.e. > 6 SD in any of the 10 principal components of variation), were also excluded. After quality control, pre-phasing of genotypes was performed using SHAPEIT2 [19] and genotype imputation using IMPUTE2. The 1000G Phase1 integrated haplotypes was used as the reference panel [20, 21].

A total of 30,038,143 markers were finally imputed from the Spain GWAS cohort. From these, after filtering for high imputation quality (info score > 0.8, *n* = 9,168,673) and minor allele frequency (MAF) > 1%), 7,195,283 markers were available for GWAS. Association testing was performed using the logistic regression model implemented in SNPTEST v2 software adjusting for the first two principal components of variation [22].

Meta-analysis of the common markers between the two GWAS datasets was performed using METAL [23]. In this approach, *z* values are computed to summarize both the direction of effect and the significance level for each genetic marker. These *z* values are then combined in a weighted sum that incorporates the sample size of each cohort. The complete results from the Spain GWAS and from the GWAS meta-analysis are available for download at http://urr.cat/data/GWAS_SLE_summaryStats.zip. Association plots for each of the associated loci were prepared using LocusZoom (http://locuszoom.org/).

### Genome-wide pathway analysis

Several approaches are actually available to perform pathway-based GWAS. However, most of these methods do not account for the linkage disequilibrium (LD) structure in the genome. The variable structure of LD, particularly of highly correlated chromosomal regions containing multiple genes, can negatively impact the results from genome-wide pathway analysis [24]. In order to integrate this information into the pathway association analysis, we used the method implemented in PASCAL [25]. In this approach, genetic markers are first mapped to genes in each pathway (here, all markers inside the gene ± 20 flanking kb). Correlated markers are then identified using the LD structure estimated from a reference population (in this study, from the Caucasian European population from the 1000 Genomes Project (1KG)). Combining the single-marker association values with the LD structure, association scores are then computed for all genes in the pathway. In those cases where genes from the same pathway are located close in a chromosome and in strong LD, a joint score is calculated. Finally, the scores of all genes within a pathway are normalized, transformed and integrated to generate a single association statistic that can be used to determine the statistical significance of the association between the pathway and the trait of interest. In this study, the default parameter values were used, including maximum number of SNPs per gene (*n* = 3000). The SNP *p* value to gene score estimation was performed using the sum gene score approach, and gene score transforming into the pathway score was performed using the chi-squared approach. The pathway analysis method implemented in PASCAL has been shown to perform better than other methods, particularly since it has better control of type I error.

Pathways and their corresponding gene annotation was obtained from the MSigDB molecular signatures repository (http://software.broadinstitute.org/gsea/msigdb). A total of 1077 biological pathways from the Reactome (*n* = 674), Kyoto Expression of Genes and Genomes (*n* = 186) and BioCarta (*n* = 217) databases were selected. The association *p* values obtained using PASCAL in the two GWAS cohorts were combined using Fisher’s method, and the significance was corrected for multiple testing using Bonferroni’s adjustment.

In order to capture biologic pathways associated with SLE through as yet unaccounted for genetic risk variants, all regions previously associated with SLE risk were removed from this analysis. For this objective, we filtered out all markers within ± 250 kb distance from an established SLE risk SNP and with an LD *r*^2^ > 0.2. Given the strong association between the HLA region and SLE risk, we excluded this region from the analysis (chr6: (bp 28,500,000–33,500,000).

## Results

### GWAS meta-analysis

After quality control, a total of 7,195,283 autosomal markers with MAF > 0.01 were available for association testing in the Spain case-control cohort. From these, 7,110,321 variants were also present in the European ancestry GWAS: 51 of the 52 previously known SLE risk loci were in the same effect-size direction as originally described. From these, 31 had nominal evidence of replication (*p <* 0.05, Additional file 1 Table S2). Four SNPs, rs1270942 (*HLA*, OR (95% CI)_Spain_ = 1.96 (1.59–2.42), OR (95% CI)_EUR_ = 2.53 (2.34–2.74), *p* value for heterogeneity (*p*_Het_) = 0.00074), rs494003 (*RNASEH2C*, OR (95% CI)_Spain_ = 1.44 (1.24–1.66), OR (95% CI)_EUR_ = 1.16 (1.07–1.26); *p*_Het_ = 0.0059), rs9652601 (*CIITA-SOCS1*, OR (95% CI)_Spain_ = 0.74 (0.66–0.85), OR (95% CI)_EUR_ = 0.85 (0.8–0.9, *p*_Het_ = 0.026), and rs3024505 (*IL10*, OR(95% CI)_Spain_ = 1.38 (1.17–1.62), OR (95% CI)_EUR_ = 1.13 (1.04–1.22); *p*_Het_ = 0.028) showed evidence of heterogeneity between the two GWAS cohorts.

**Fig. 1:**
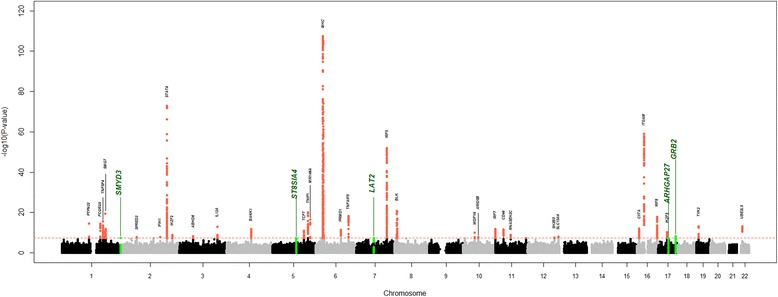
Manhattan plot of genome-wide association study meta-analysis results including the Spain cohort. Plot of the -log10 (*p* values) of association between the 7,110,321 markers after meta-analysis between the European and Spain cohorts. The dashed horizontal line represents the genome-wide significance threshold (*p* value = 5 × 10^− 8^). The known regions associated with systemic lupus erythematosus (SLE) risk and genome-wide significant are colored in red. The five new genomic regions associated with SLE risk in this study are colored in green, with the name of the corresponding gene above

**Fig. 2:**
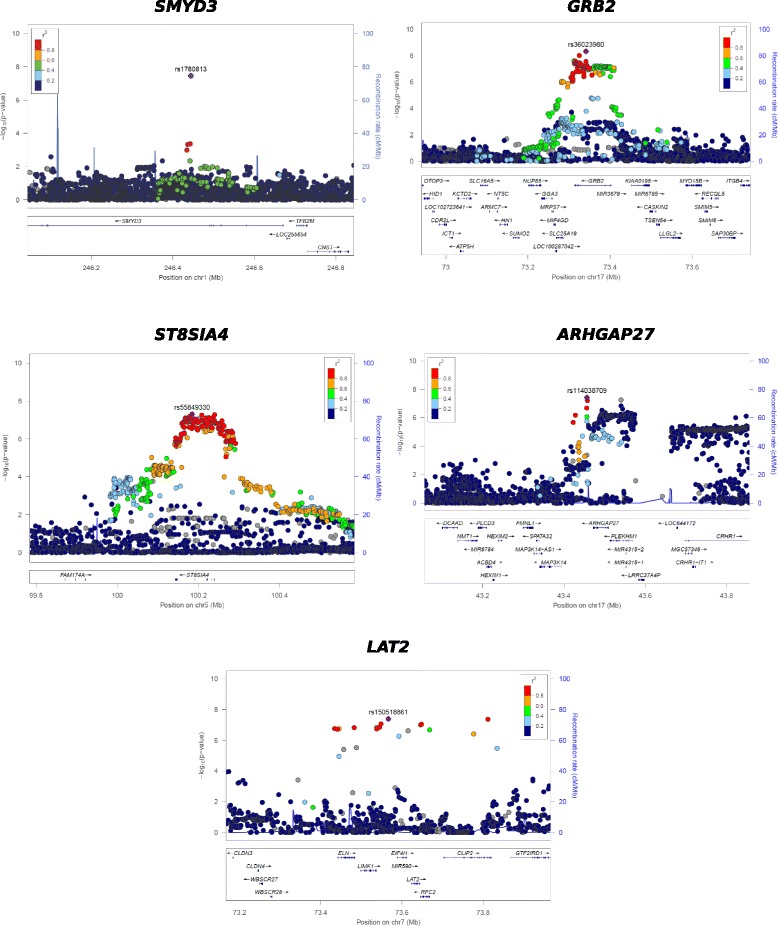
Regional association plots from the meta-analysis of the two cohorts for all five genome-wide significant loci: -log10 (*p* values) for both directly genotyped and imputed single nucleotide polymorphisms (SNPs) are plotted as a function of genomic position (NCBI Build 37). The purple diamond indicates the lead SNP at each locus; the remaining markers are colored based on the LD (*r* [2]) in relation to the lead SNP. Underlying the image, the estimated recombination rate (cM/Mb) for the CEU panel from 1000 Genomes is depicted

Meta-analysis of the two GWAS cohorts identified five new risk loci for SLE (Fig. 1, Table 1). None of the new genome-wide significant loci showed evidence of heterogeneity of effect between the two cohorts (*p* > 0.05). Three of the associated markers are SNPs in introns of the genes encoding for growth factor receptor bound protein 2 (*GRB2,* rs36023980), SET and MYND domain containing 3 protein (*SMYD3,* rs1780813), and ST8 alpha-N-acetyl-neuraminide alpha-2,8-sialyltransferase 4 (*ST8SIA4,* rs55849330). Associated SNPs rs150518861 and rs114038709 are located in the flanking regions of linker for activation of T cells family member 2 (*LAT2*) and Rho GTPase activating protein 2 (*ARHGAP27*) genes, respectively. Figure 2 shows the detailed association results for each of the five new SLE risk loci.

**Table 1:** Novel SNPs for SLE risk showing genome-wide significance (*p <* 5 × 10^− 8^) following meta-analysis of Spain and European ancestry cohorts

					European		Spain		Meta-analysis	
Locus	Chr	SNP	bp	RA	OR (CI 95%)	*p* value	OR (CI 95%)	*p* value	OR (CI 95%)	*p* value
*SMYD3*	1	rs1780813	246,444,082	*C*	0.53 (0.40–0.69)	8.36 × 10^−7^	0.61 (0.37–0.98)	0.013	0.55 (0.31–0.79)	3.5 × 10^−8^
*ST8SIA4*	5	rs55849330	100,184,647	*A*	1.16 (1.10–.123)	8.4 × 10^−7^	1.14 (1.01–1.30)	0.019	1.16 (1.11–1.21)	4.9 × 10^−8^
*LAT2*	7	rs150518861	73,566,677	*A*	1.63 (1.34–1.99)	0.0000015	1.77 (1.23–2.56)	0.0074	1.66 (1.49–1.84)	4.1 × 10^−8^
*ARHGAP27*	17	rs114038709	43,456,728	*T*	1.15 (1.08–1.22)	0.0000012	1.20 (1.07–1.35)	0.0088	1.16 (1.11–1.22)	3.7 × 10^−8^
*GRB2*	17	rs36023980	73,341,284	*C*	1.18(1.11–1.25)	0.0000015	1.23 (1.08–1.40)	0.00039	1.18 (1.13–1.24)	4.7 × 10^−9^

OR are shown for the minor allele for all five associated polymorphisms

*Locus* closest gene, *Chr* chromosome, *SNP* single nucleotide polymorphism, *bp* base pair, *RA* risk allele, *OR* odds ratio

### Genetic pathway association study in SLE

After excluding the association signal from known risk loci, pathway analysis identified 100 and 157 pathways associated with SLE in the Spain and European-ancestry cohorts, respectively, at the nominal level (*p* < 0.05). From these, 30 pathways (3% of total) were found to be associated in both cohorts, which is more than would be expected by chance (*p* < 5 × 10^− 4^). After adjustment for multiple testing, four biologic pathways were significantly associated with SLE risk (Table 2).

**Table 2:** Biological pathways significantly associated with SLE risk

Biological pathway	N genes	Spain cohort *p* value	European cohort *p* value	Combined *p* value	Adjusted *p* value
B cell receptor signaling	75	0.016	2.05 × 10^−6^	5.28 × 10^− 6^	0.0057
CTLA4 co-stimulatory signal during T-cell activation	21	0.0014	0.0016	3.06 × 10^−5^	0.033
Interleukin-4 signaling	11	0.00029	0.01	3.97 × 10^−5^	0.043
Cell surface interactions at the vascular wall	91	0.0057	0.0006	4.63 × 10^−5^	0.049

Biological pathways significantly associated with systemic lupus erythematosus (SLE) after meta-analysis of the Spain and Caucasian European cohorts. *P* values for each cohort were estimated using PASCAL after removing the previously known risk loci for SLE

*N genes* number of genes in the pathway

### Conditional and sex-specific association

In order to explore the presence of secondary signals at each associated locus, we performed conditional analysis in the Spain cohort. From all five loci, we only identified one independent signal within 1 Mb of the most strongly associated SNP that continued to show evidence of association (conditional *p* < 1 × 10^− 4^). This independent association was identified at the *GRB2* locus and maps to *RNF157* gene (SNP rs9891273, *p* = 4.99 × 10^− 5^, Additional file 1: Figure S3).

The presence of sex-specific associations was tested by comparing the coefficients for SNP association estimated independently in women and men. We identified a significant difference only for *LAT2* SNP rs150518861 (*p* = 0.032). This risk variant was found to be more strongly associated in the male cohort compared to the female cohort.

## Discussion

In the present study we have identified five new risk loci for systemic lupus erythematosus. Performing a meta-analysis on 4943 patients with SLE and 8483 controls from different European ancestries, we have identified variants at *GRB2, SMYD3*, *ST8SIA4*, *LAT2*, and *ARHGAP27* loci associated with SLE susceptibility. At the pathway level, we have also found four biological pathways associated with SLE risk independently of previously known risk genes.

In the present meta-analysis, we found an association between an intronic SNP in the gene encoding for the growth factor receptor-bound protein GRB2 and SLE (rs36023980, *p* = 4.7 × 10^− 9^). Analysis of the tissue-specific epigenetic data from the NIH Roadmap Epigenomics Project [26] for rs36023980 SNP showed a strong regulatory activity in different immune cells, including enhancer evidence in both T and B lymphocytes (Additional file 1: Table S2). *GRB2* encodes for a receptor tyrosine-kinase (RTK) adaptor protein composed of a single SH2 domain and two SH3 domains [27]. SLE is a disease characterized by the activation of B cells that recognize self-antigens via their B cell receptors (BCR). In B cells, GRB2 functions as an expression adaptor molecule, attenuating the signals that are transduced by the BCR [28]. Together with Dok-3 and SHIP1, GRB2 forms a trimer protein complex that binds directly to the BCR and prevents downstream signaling by inhibiting PI3K signaling [29]. Gene expression at different stages of B cell differentiation shows that *GRB2* expression increases in more mature forms, particularly on antigen-experienced memory B cells (Additional file 1: Figure S4). Inadequate control of memory B cell differentiation into plasma cells has been proposed as a trigger for autoimmunity in SLE [30]. Our results therefore are in line with the relevance of this causal disease mechanism.

In a close functional relationship with *GRB2*, we also found a significant association between linker for activation of T cells family member 2 gene (*LAT2*) locus and SLE (rs150518861, *p* = 4.1 × 10^− 8^). *LAT2* encodes for an adaptor molecule that binds GRB2 and, therefore, is also involved in BCR signaling [31]. The association at the genetic level between SLE and two directly interacting proteins strongly supports the implication of this biological mechanism in SLE risk. B cell dysfunction is a hallmark of SLE pathology [9], and our study supports downstream regulation after antigen binding as a crucial event in the disease etiology. In the evaluation of sex-specific effects, we found this locus to be differentially associated with SLE risk. The risk variant was associated with SLE in men (*p* = 0.0074, β (95% CI) = 1.3 (0.25–2.2)), and it was non-significant in women (*p* = 0.58, β (95% CI) = 0.13 (− 0.33 to 0.62)). Previous studies have shown that men require a higher genetic load to develop the disease [32]. If replicated in an independent cohort, this result would be in line with these findings, confirming the importance of sex in mediating the effect of some genetic risk factors in SLE. *SMYD3* encodes for an H3-Hk histone methyltransferase that has been associated with increased cell proliferation in cancer [33]. Altered epigenetic patterns have been strongly associated with SLE, mostly at the DNA level [34]. More recently, however, methylated histones have also been identified as targets of autoantibodies expressed in patients with SLE [35]. Similar to other frequent nuclear autoantigens in SLE, like double-stranded DNA or ribonucleoproteins, methylated H3-Hk histones are able to trigger autoreactive B cells after antigenic-exposure processes like apoptosis. According to the Roadmap Epigenomics Project data, the associated SNP rs1780813 lies in a site that is DNAse hypersensitive for > 30 different tissues, supporting its role in gene regulation.

To date little is known about the functional role of SLE-associated genes *STS8IA4* and *ARHGAP27*. In order to infer the potential biological role of these two genes, we used the GeneNetwork approach, a functional-inference method based on the gene co-expression patterns extracted from microarray data from > 80,000 samples [36]. With this approach, we found strong evidence that *STS8IA4* is involved in T cell activation (*p* value = 2.7 × 10^− 13^, Additional file 2: Table S4), and that *ARHGAP27* is implicated in mitogen-activated protein kinase (MAPKinase) signaling (*p* value = 3.33 × 10^− 8^, Additional file 2: Table S5). Both biological processes have been previously associated with SLE etiology, and our results not only support their involvement in disease risk but also suggest new gene functions. Furthermore, expression quantitative trait locus (eQTL) evidence supports that both SNPs regulate expression of the corresponding genes in *cis*. Whole blood eQTL analysis [37] shows a strong association between variation at rs114038709 and *ARHGAP27* expression (*p* = 4.1 × 10^− 134^), and the most significant eQTL evidence for rs55849330 is associated to *STS8IA4* expression in immortalized B cells [38] (*p* = 5.6 × 10^− 10^).

Using a pathway-based analysis we have identified four biological pathways associated with SLE. Since the objective was to identify new genetic risk variation for SLE, our approach excluded all association signals from previously known SLE genes. We showed that by using biological pathway knowledge, it is still possible to capture genetic variation that is relevant for the disease. One limitation of this approach is that it relies on the specific knowledge of gene functions and pathway definitions, which is still relatively low for a substantial fraction of the genome [39]. Another limitation is that pathway association is performed on variants within or close to genes. Distant *cis* regulation and also *trans* regulation variants are also plausible mechanisms of action [40]. With better knowledge of regulatory effects, particularly on isolated cell types, pathway-based analysis will become an even more powerful approach to detect the missing disease heritability. Despite these limitations, our results are robust since they are based on strongly supported biological knowledge. Also, we provide statistical evidence of pathway association from two independent GWAS cohorts which, to our knowledge, has not been previously performed in SLE.

The BCR signaling pathway had the strongest association with SLE. This result is in agreement with the results found at the single-marker level, where variants at BCR signaling genes *GRB2* and *LAT2* were found to be associated with disease susceptibility. Within the BCR signaling pathway, however, there are multiple other single-marker hits in other genes indicating nominally significant association with disease susceptibility in both cohorts. Given that they belong to an associated biological pathway, these signals are strongly suggestive risk variants for SLE (Table 3). Of relevance, several of the proteins encoded by the genes in this pathway, like *BTK* or *CTLA4*, are currently being evaluated as therapeutic targets for SLE [41, 42]. Finding an efficacious treatment in SLE has proven extremely difficult and our results support the importance of targeting this pathway. Genetic evidence, either direct or through associated gene networks, has been shown to improve drug efficacy prediction [43]. Based on the association signals found in the two cohorts, for example, the proteins encoded by *LYN* (*p* = 1.17 × 10^− 6^) and *NFATC1* (*p* = 5.26 × 10^− 6^) could also be considered as new drug targets for SLE.

**Table 3:** Top single-marker hits in genes from the four genetic pathways associated with SLE

Gene	Marker	Chr	bp	MA	OR	*p* (Spain)	*p* (EUR)	*p* (meta)	Pathway
*BCL10*	rs12084253	1	85,720,326	T	1.11	0.020	0.0015	0.00012	BCR
*FCER1G*	rs1136224	1	161,184,097	G	0.91	0.023	0.022	0.0024	VASC
*FCGR2B*	rs182968886	1	161,642,985	A	0.86	0.044	0.0018	0.00023	BCR
*CD247*	rs113305799	1	167,416,006	A	1.17	0.0035	0.044	0.0022	CTLA4
*PROC*	rs6740067	2	128,156,366	T	1.16	0.043	0.018	0.0026	VASC
*CTLA4*	rs733618	2	204,730,944	C	1.19	0.026	0.0018	0.00016	CTLA4
*PPP3CA*	rs13120190	4	102,056,663	G	0.93	0.025	0.047	0.0060	BCR
*IL2*	rs45522533	4	123,396,876	T	1.16	0.016	0.0058	0.00044	CTLA4
*SLC7A11*	rs74843273	4	139,150,464	T	0.81	0.025	0.0066	0.00065	VASC
*ITK*	rs60714766	5	156,602,589	T	1.07	0.015	0.043	0.0042	CTLA4
*CARD11*	rs6461796	7	3,071,195	C	0.94	0.027	0.033	0.0042	BCR
*LYN*	rs17812659	8	56,889,862	G	0.86	0.013	2.57E-05	1.17E-06	BCR,VASC
*ANGPT1*	rs79847080	8	108,293,443	G	0.84	0.032	0.0028	0.00031	VASC
*VAV2*	rs2810536	9	136,812,625	G	1.08	0.030	0.011	0.0013	BCR
*KRAS*	rs17388587	12	25,389,220	G	1.13	0.036	0.048	0.0075	BCR, VASC
*PRKCB*	rs11641223	16	24,020,316	T	1.11	0.041	0.0010	0.00012	BCR
*CD19*	16:28955702:D	16	28,955,702	I	1.06	0.0077	0.047	0.0034	BCR
*SLC7A6*	rs55856208	16	68,324,210	T	1.08	0.045	0.049	0.0086	VASC
*PLCG2*	rs11548656	16	81,916,912	G	1.3	0.014	0.00062	0.000035	BCR
*ATP1B2*	rs1794287	17	7,578,837	A	0.9	0.023	0.024	0.0026	VASC
*ITGB3*	rs75211989	17	45,366,261	G	1.11	0.00014	0.020	0.00020	VASC
*GRB2*	rs36023980	17	73,341,284	T	0.85	0.00039	1.51E-06	4.73E-09	CTLA4, IL4, BCR, VASC
*NFATC1*	rs111354805	18	77,238,078	T	1.21	0.027	6.58E-05	5.26E-06	BCR
*MAP2K2*	rs350913	19	4,096,779	T	0.94	0.029	0.030	0.0039	BCR
*CD79A*	rs16975619	19	42,392,441	C	1.52	0.020	0.0099	0.00089	BCR
*SIRPG*	rs11696739	20	1,600,925	A	0.92	0.044	0.0050	0.00069	VASC
*RAC2*	rs229566	22	37,602,131	A	1.06	0.041	0.03	0.0047	BCR

Suggestive risk variants were identified as those markers showing with the most significant meta-analysis *p* value (*p* (meta)), and that are associated in the two genome-wide association study cohorts (*p <* 0.05) and show the same direction of effect (OR) *MA* minor allele, *OR* odds ratio according to minor allele in European ancestry cohort, *I* insertion allele, *Pathway* biological pathway/s where the gene has been annotated, *BCR* B cell receptor pathway, *CTLA4* CTLA4 pathway, *IL4* interleukin-4 pathway, *VASC* vascular cell wall pathway

Two other associated pathways - the CTLA4 co-stimulatory signal and IL4 pathways - are strongly related to B cell activation. CTLA4 is a co-inhibitory molecule expressed on activated helper T - TH2 and follicular - cells. Inhibition of CTLA4 increases B cell activation after antigen binding, resulting in the production of antibodies [44]. IL-4 is a cytokine that is also expressed in helper T cells and it is essential in the activation of antigen-bound naïve B cells. Similar to the BCR signaling pathway, these two genetically associated biological processes that are deeply related to B cell activation could be the source of new effective drug targets for the disease [45]. In this regard, a fusion protein including the extracellular domain of CTLA4 (abatacept) is being currently evaluated as a therapy for more severe forms of SLE [46].

## Conclusions

In the present study we have performed a GWAS meta-analysis approach to identify new genetic variation in SLE. We have found five new genome-wide significant risk loci and four biologic pathways associated with SLE risk. Single-marker associations involve BCR downstream signaling mechanisms with disease susceptibility, and autoantigen generation and immune cell activity regulation. Pathway-based analysis confirmed the relevance of BCR signaling pathway and other B cell activation mechanisms in the disease etiology. The results from this study significantly expand the knowledge of the biological processes implicated in susceptibility to SLE.

## Additional files

Additional file 1: **Table S1.** Epidemiological features from the Spain GWAS cohort. **Figure S1.** Principal component analysis of the Spain GWAS cohort. **Figure S2.** Quantile-quantile(Q-Q) plots of observed and expected -log10(*p* values) of association between SNP genotype and SLE risk. **Table S2.** Epigenetic regulatory data associated with *GRB2* risk locus. **Figure S3.** Regional association plot for the association with SLE risk independent of *GRB2* SNP rs36023980. **Figure S4.** *GRB2* gene expression during human B cell differentiation. (DOCX 627 kb)

Additional file 2: **Table S3.** Association results for the 52 previously known SLE risk loci in the Spain GWAS. **Table S4.** Pathway association results after combining the two SLE cohorts (combined raw *p* value <0.05). **Table S5.** List of biological pathways significantly associated with ST8SIA4 gene network. **Table S6.** List of biological pathways significantly associated with ARHGAP27 gene network. (XLSX 48 kb)

## Abbreviations

BCR: B cell receptor
bp: Base pair
CI: Confidence interval
eQTL: Expression quantitative trait locus
GWAS: Genome-wide association study
HLA: Human leukocyte antigen
IMID Consortium: Immune-Mediated Inflammatory Disease Consortium
LD: Linkage disequilibrium
MAF: Minor allele frequency
OR: Odds ratio
*p*_Het_: *P* value for heterogeneity on genetic effect
SLE: Systemic lupus erythematosus
SNP: Single nucleotide polymorphism
