# Supplementary material for: Genome-wide association study meta-analysis identifies five new loci for systemic lupus erythematosus
Source: Arthritis Res Ther. 2018 May 30;20:100. doi: 10.1186/s13075-018-1604-1 (PMC5977506; doi:10.1186/s13075-018-1604-1)

**SUPPLEMENTARY FIGURES AND TABLES**

#

# Table S1. Epidemiological features from the Spain GWAS cohort.

|  | SLE cohort (n=907) | Control Cohort (n=1,524) |
| --- | --- | --- |
| Caucasian European | 100% | 100% |
| Female, n (%) | 841 (92.7%) | 610 (40%) |
| Age (mean ± SD) | 45.7±13.9 | 49.6±7.0 |
| Age at onset (mean ± SD) | 32.9±13.7 | - |

**Figure S1. Principal Component Analysis of the Spain GWAS cohort.** **A** Plots of the principal components of variation of the Spain. cohort in relation to the Hapmap Caucasian European (blue), African Yoruba (orange) and Asian Han Chinese and Japanese populations (red). As expected, the Spanish case-control cohort first and second PCs are closer to the CEU cohort (Northern European ancestry), although they don’t overlap. **B**. A closer view of the first and second PCs for the Spain GWAS show the total overlap between SLE patients (green) and healthy controls (grey), after excluding outlier individuals (i.e. patients or controls at >6 standard deviations from any of the 10 main PCs).

**A**


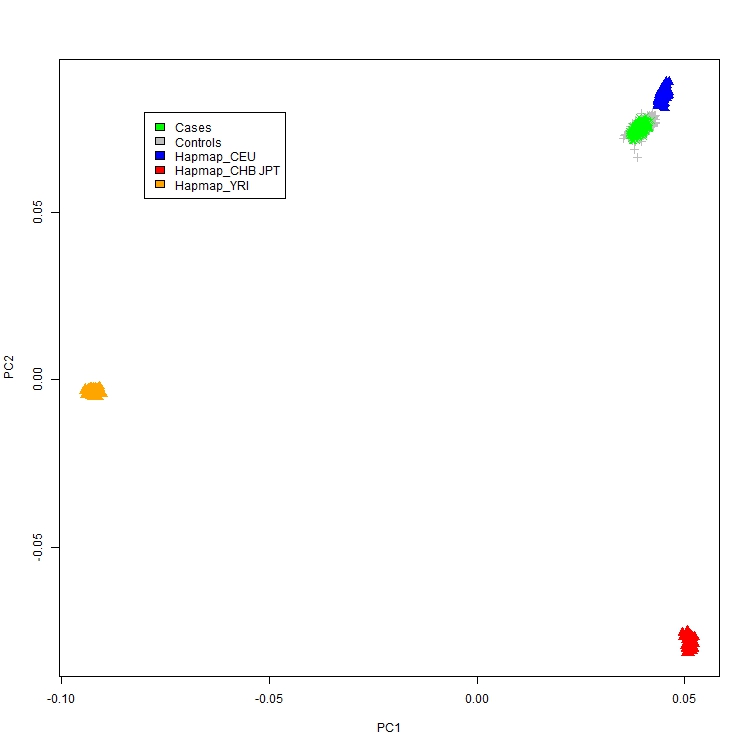


**B**


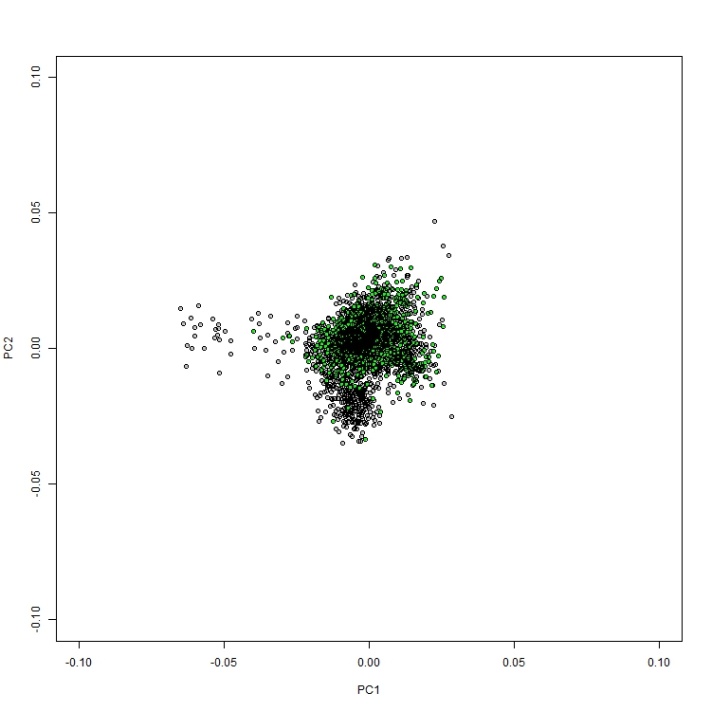


# Figure S2. Quantile-Quantile(Q-Q) plots of observed and expected -log10(p-values) of association between SNP genotype and SLE risk. (A) European ancestry (EUR) and (B) Spain cohort.

1. EUR (λ_GC_ = 1.16)


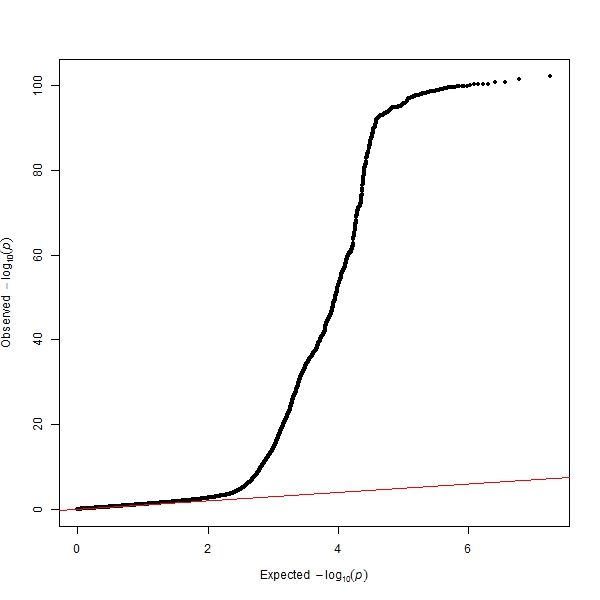


1. Spain (λ_GC_ = 1.06)


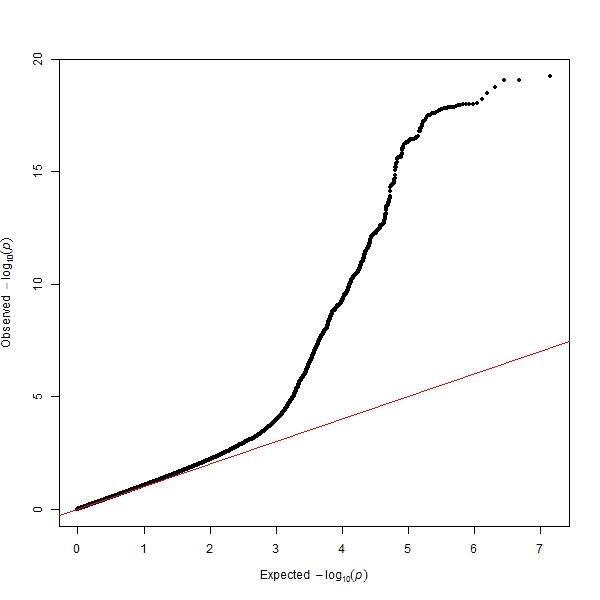


# Table S2. Epigenetic regulatory associated with *GRB2* risk locus. Functional regulatory data for associated *GRB2* SNP rs36023980 was obtained from Haploreg v.4.1 (<http://archive.broadinstitute.org/mammals/haploreg/haploreg.php>). DNase I hypersensitivity and ChIP-Seq data generated from the Roadmap Epigenomics Consortium (2015) on multiple cell lines and human tissues analyzed for enrichment. The data for this SNP strongly supports a regulatory activity, including enhancer activity for different immune cells including T and B lymphocytes. Color coding is as follows: green, weak transcription; yellow and orange, enhancer; red, promoter; blue, insulator; gray, repressed.

|  |  |  |  |  |  |  |  |  |  |  |
| --- | --- | --- | --- | --- | --- | --- | --- | --- | --- | --- |
| **Epigenome ID (EID)** | **Group** | **Mnemonic** | **Description** | **Chromatin states (Core 15-state model)** | **Chromatin states (25-state model using 12 imputed marks)** | **H3K4me1** | **H3K4me3** | **H3K27ac** | **H3K9ac** | **DNase** |
| E017 | IMR90 | LNG.IMR90 | IMR90 fetal lung fibroblasts Cell Line |  |  | H3K4me1_Enh |  |  |  |  |
| E002 | ESC | ESC.WA7 | ES-WA7 Cells |  |  |  |  |  |  |  |
| E008 | ESC | ESC.H9 | H9 Cells | 1_TssA | 10_TxEnh5 | H3K4me1_Enh | H3K4me3_Pro | H3K27ac_Enh |  |  |
| E001 | ESC | ESC.I3 | ES-I3 Cells | 7_Enh | 4_PromD2 | H3K4me1_Enh | H3K4me3_Pro |  | H3K9ac_Pro |  |
| E015 | ESC | ESC.HUES6 | HUES6 Cells | 1_TssA | 9_TxReg | H3K4me1_Enh | H3K4me3_Pro | H3K27ac_Enh | H3K9ac_Pro |  |
| E014 | ESC | ESC.HUES48 | HUES48 Cells | 7_Enh | 4_PromD2 | H3K4me1_Enh | H3K4me3_Pro | H3K27ac_Enh | H3K9ac_Pro |  |
| E016 | ESC | ESC.HUES64 | HUES64 Cells | 1_TssA | 13_EnhA1 | H3K4me1_Enh | H3K4me3_Pro | H3K27ac_Enh | H3K9ac_Pro |  |
| E003 | ESC | ESC.H1 | H1 Cells | 7_Enh | 9_TxReg | H3K4me1_Enh | H3K4me3_Pro | H3K27ac_Enh | H3K9ac_Pro |  |
| E024 | ESC | ESC.4STAR | ES-UCSF4 Cells | 7_Enh | 18_EnhAc | H3K4me1_Enh |  |  |  |  |
| E020 | iPSC | IPSC.20B | iPS-20b Cells | 1_TssA | 14_EnhA2 | H3K4me1_Enh | H3K4me3_Pro | H3K27ac_Enh | H3K9ac_Pro |  |
| E019 | iPSC | IPSC.18 | iPS-18 Cells | 1_TssA | 4_PromD2 | H3K4me1_Enh | H3K4me3_Pro | H3K27ac_Enh | H3K9ac_Pro |  |
| E018 | iPSC | IPSC.15b | iPS-15b Cells | 7_Enh | 22_PromP | H3K4me1_Enh | H3K4me3_Pro |  | H3K9ac_Pro |  |
| E021 | iPSC | IPSC.DF.6.9 | iPS DF 6.9 Cells |  | 18_EnhAc | H3K4me1_Enh |  | H3K27ac_Enh |  |  |
| E022 | iPSC | IPSC.DF.19.11 | iPS DF 19.11 Cells |  |  |  |  | H3K27ac_Enh |  |  |
| E007 | ES-deriv | ESDR.H1.NEUR.PROG | H1 Derived Neuronal Progenitor Cultured Cells |  |  |  |  |  |  |  |
| E009 | ES-deriv | ESDR.H9.NEUR.PROG | H9 Derived Neuronal Progenitor Cultured Cells |  |  | H3K4me1_Enh |  |  |  |  |
| E010 | ES-deriv | ESDR.H9.NEUR | H9 Derived Neuron Cultured Cells | 7_Enh | 18_EnhAc | H3K4me1_Enh |  |  |  |  |
| E013 | ES-deriv | ESDR.CD56.MESO | hESC Derived CD56+ Mesoderm Cultured Cells |  |  | H3K4me1_Enh |  | H3K27ac_Enh |  |  |
| E012 | ES-deriv | ESDR.CD56.ECTO | hESC Derived CD56+ Ectoderm Cultured Cells |  | 18_EnhAc | H3K4me1_Enh |  | H3K27ac_Enh |  |  |
| E011 | ES-deriv | ESDR.CD184.ENDO | hESC Derived CD184+ Endoderm Cultured Cells | 7_Enh | 18_EnhAc | H3K4me1_Enh | H3K4me3_Pro | H3K27ac_Enh |  |  |
| E004 | ES-deriv | ESDR.H1.BMP4.MESO | H1 BMP4 Derived Mesendoderm Cultured Cells | 7_Enh | 18_EnhAc | H3K4me1_Enh |  | H3K27ac_Enh |  |  |
| E005 | ES-deriv | ESDR.H1.BMP4.TROP | H1 BMP4 Derived Trophoblast Cultured Cells |  |  | H3K4me1_Enh |  |  |  |  |
| E006 | ES-deriv | ESDR.H1.MSC | H1 Derived Mesenchymal Stem Cells |  |  | H3K4me1_Enh |  |  |  |  |
| E062 | Blood & T-cell | BLD.PER.MONUC.PC | Primary mononuclear cells from peripheral blood | 7_Enh | 10_TxEnh5 | H3K4me1_Enh | H3K4me3_Pro | H3K27ac_Enh | H3K9ac_Pro |  |
| E034 | Blood & T-cell | BLD.CD3.PPC | Primary T cells from peripheral blood | 7_Enh | 10_TxEnh5 | H3K4me1_Enh |  | H3K27ac_Enh |  |  |
| E045 | Blood & T-cell | BLD.CD4.CD25I.CD127.TMEMPC | Primary T cells effector/memory enriched from peripheral blood | 7_Enh | 10_TxEnh5 | H3K4me1_Enh |  | H3K27ac_Enh |  |  |
| E033 | Blood & T-cell | BLD.CD3.CPC | Primary T cells from cord blood | 7_Enh | 10_TxEnh5 | H3K4me1_Enh | H3K4me3_Pro |  |  |  |
| E044 | Blood & T-cell | BLD.CD4.CD25.CD127M.TREGPC | Primary T regulatory cells from peripheral blood | 7_Enh | 10_TxEnh5 | H3K4me1_Enh | H3K4me3_Pro | H3K27ac_Enh |  |  |
| E043 | Blood & T-cell | BLD.CD4.CD25M.TPC | Primary T helper cells from peripheral blood | 7_Enh | 10_TxEnh5 | H3K4me1_Enh | H3K4me3_Pro | H3K27ac_Enh |  |  |
| E039 | Blood & T-cell | BLD.CD4.CD25M.CD45RA.NPC | Primary T helper naive cells from peripheral blood | 7_Enh | 9_TxReg | H3K4me1_Enh | H3K4me3_Pro | H3K27ac_Enh |  |  |
| E041 | Blood & T-cell | BLD.CD4.CD25M.IL17M.PL.TPC | Primary T helper cells PMA-I stimulated | 7_Enh | 9_TxReg | H3K4me1_Enh | H3K4me3_Pro | H3K27ac_Enh |  |  |
| E042 | Blood & T-cell | BLD.CD4.CD25M.IL17P.PL.TPC | Primary T helper 17 cells PMA-I stimulated | 7_Enh | 10_TxEnh5 | H3K4me1_Enh | H3K4me3_Pro | H3K27ac_Enh |  |  |
| E040 | Blood & T-cell | BLD.CD4.CD25M.CD45RO.MPC | Primary T helper memory cells from peripheral blood 1 | 7_Enh | 10_TxEnh5 | H3K4me1_Enh | H3K4me3_Pro | H3K27ac_Enh |  |  |
| E037 | Blood & T-cell | BLD.CD4.MPC | Primary T helper memory cells from peripheral blood 2 | 7_Enh | 10_TxEnh5 | H3K4me1_Enh | H3K4me3_Pro | H3K27ac_Enh |  |  |
| E048 | Blood & T-cell | BLD.CD8.MPC | Primary T CD8+ memory cells from peripheral blood | 7_Enh | 10_TxEnh5 | H3K4me1_Enh | H3K4me3_Pro |  |  |  |
| E038 | Blood & T-cell | BLD.CD4.NPC | Primary T helper naive cells from peripheral blood | 7_Enh | 10_TxEnh5 | H3K4me1_Enh | H3K4me3_Pro | H3K27ac_Enh | H3K9ac_Pro |  |
| E047 | Blood & T-cell | BLD.CD8.NPC | Primary T CD8+ naive cells from peripheral blood | 7_Enh | 10_TxEnh5 | H3K4me1_Enh |  | H3K27ac_Enh |  |  |
| E029 | HSC & B-cell | BLD.CD14.PC | Primary monocytes from peripheral blood | 7_Enh | 9_TxReg | H3K4me1_Enh |  | H3K27ac_Enh |  |  |
| E031 | HSC & B-cell | BLD.CD19.CPC | Primary B cells from cord blood | 7_Enh | 9_TxReg | H3K4me1_Enh | H3K4me3_Pro |  |  |  |
| E035 | HSC & B-cell | BLD.CD34.PC | Primary hematopoietic stem cells | 7_Enh | 10_TxEnh5 | H3K4me1_Enh |  |  |  |  |
| E051 | HSC & B-cell | BLD.MOB.CD34.PC.M | Primary hematopoietic stem cells G-CSF-mobilized Male | 7_Enh | 10_TxEnh5 | H3K4me1_Enh |  |  |  |  |
| E050 | HSC & B-cell | BLD.MOB.CD34.PC.F | Primary hematopoietic stem cells G-CSF-mobilized Female | 7_Enh | 9_TxReg | H3K4me1_Enh |  | H3K27ac_Enh |  |  |
| E036 | HSC & B-cell | BLD.CD34.CC | Primary hematopoietic stem cells short term culture | 7_Enh | 9_TxReg | H3K4me1_Enh | H3K4me3_Pro |  |  |  |
| E032 | HSC & B-cell | BLD.CD19.PPC | Primary B cells from peripheral blood | 2_TssAFlnk | 9_TxReg | H3K4me1_Enh | H3K4me3_Pro | H3K27ac_Enh |  |  |
| E046 | HSC & B-cell | BLD.CD56.PC | Primary Natural Killer cells from peripheral blood | 7_Enh | 9_TxReg | H3K4me1_Enh |  | H3K27ac_Enh |  |  |
| E030 | HSC & B-cell | BLD.CD15.PC | Primary neutrophils from peripheral blood | 7_Enh | 9_TxReg | H3K4me1_Enh | H3K4me3_Pro |  |  |  |
| E026 | Mesench | STRM.MRW.MSC | Bone Marrow Derived Cultured Mesenchymal Stem Cells |  |  |  |  |  |  |  |
| E049 | Mesench | STRM.CHON.MRW.DR.MSC | Mesenchymal Stem Cell Derived Chondrocyte Cultured Cells |  |  |  |  |  |  |  |
| E025 | Mesench | FAT.ADIP.DR.MSC | Adipose Derived Mesenchymal Stem Cell Cultured Cells |  |  |  |  |  |  |  |
| E023 | Mesench | FAT.MSC.DR.ADIP | Mesenchymal Stem Cell Derived Adipocyte Cultured Cells |  |  |  |  |  |  |  |
| E052 | Myosat | MUS.SAT | Muscle Satellite Cultured Cells |  |  | H3K4me1_Enh |  |  |  |  |
| E055 | Epithelial | SKIN.PEN.FRSK.FIB.01 | Foreskin Fibroblast Primary Cells skin01 |  |  | H3K4me1_Enh |  | H3K27ac_Enh |  |  |
| E056 | Epithelial | SKIN.PEN.FRSK.FIB.02 | Foreskin Fibroblast Primary Cells skin02 |  |  |  |  |  |  |  |
| E059 | Epithelial | SKIN.PEN.FRSK.MEL.01 | Foreskin Melanocyte Primary Cells skin01 |  |  |  |  |  |  |  |
| E061 | Epithelial | SKIN.PEN.FRSK.MEL.03 | Foreskin Melanocyte Primary Cells skin03 |  |  | H3K4me1_Enh |  |  |  |  |
| E057 | Epithelial | SKIN.PEN.FRSK.KER.02 | Foreskin Keratinocyte Primary Cells skin02 |  |  | H3K4me1_Enh |  |  |  |  |
| E058 | Epithelial | SKIN.PEN.FRSK.KER.03 | Foreskin Keratinocyte Primary Cells skin03 |  |  |  |  |  |  |  |
| E028 | Epithelial | BRST.HMEC.35 | Breast variant Human Mammary Epithelial Cells (vHMEC) |  |  | H3K4me1_Enh |  |  |  |  |
| E027 | Epithelial | BRST.MYO | Breast Myoepithelial Primary Cells |  |  |  |  |  |  |  |
| E054 | Neurosph | BRN.GANGEM.DR.NRSPHR | Ganglion Eminence derived primary cultured neurospheres |  |  | H3K4me1_Enh |  |  |  |  |
| E053 | Neurosph | BRN.CRTX.DR.NRSPHR | Cortex derived primary cultured neurospheres | 7_Enh |  | H3K4me1_Enh |  |  |  |  |
| E112 | Thymus | THYM | Thymus | 2_TssAFlnk | 9_TxReg | H3K4me1_Enh | H3K4me3_Pro | H3K27ac_Enh |  |  |
| E093 | Thymus | THYM.FET | Fetal Thymus | 7_Enh | 9_TxReg | H3K4me1_Enh | H3K4me3_Pro | H3K27ac_Enh |  |  |
| E071 | Brain | BRN.HIPP.MID | Brain Hippocampus Middle |  |  | H3K4me1_Enh |  |  |  |  |
| E074 | Brain | BRN.SUB.NIG | Brain Substantia Nigra |  |  | H3K4me1_Enh |  | H3K27ac_Enh |  |  |
| E068 | Brain | BRN.ANT.CAUD | Brain Anterior Caudate | 7_Enh |  | H3K4me1_Enh |  | H3K27ac_Enh | H3K9ac_Pro |  |
| E069 | Brain | BRN.CING.GYR | Brain Cingulate Gyrus |  |  | H3K4me1_Enh |  | H3K27ac_Enh |  |  |
| E072 | Brain | BRN.INF.TMP | Brain Inferior Temporal Lobe |  |  |  |  | H3K27ac_Enh |  |  |
| E067 | Brain | BRN.ANG.GYR | Brain Angular Gyrus |  |  | H3K4me1_Enh |  |  |  |  |
| E073 | Brain | BRN.DL.PRFRNTL.CRTX | Brain_Dorsolateral_Prefrontal_Cortex |  |  | H3K4me1_Enh |  |  |  |  |
| E070 | Brain | BRN.GRM.MTRX | Brain Germinal Matrix | 7_Enh |  | H3K4me1_Enh |  |  |  |  |
| E082 | Brain | BRN.FET.F | Fetal Brain Female |  |  | H3K4me1_Enh |  |  |  |  |
| E081 | Brain | BRN.FET.M | Fetal Brain Male | 7_Enh |  | H3K4me1_Enh |  |  |  |  |
| E063 | Adipose | FAT.ADIP.NUC | Adipose Nuclei | 7_Enh |  | H3K4me1_Enh |  | H3K27ac_Enh | H3K9ac_Pro |  |
| E100 | Muscle | MUS.PSOAS | Psoas Muscle |  |  | H3K4me1_Enh |  | H3K27ac_Enh |  |  |
| E108 | Muscle | MUS.SKLT.F | Skeletal Muscle Female | 7_Enh |  | H3K4me1_Enh |  |  |  |  |
| E107 | Muscle | MUS.SKLT.M | Skeletal Muscle Male | 7_Enh |  | H3K4me1_Enh |  |  |  |  |
| E089 | Muscle | MUS.TRNK.FET | Fetal Muscle Trunk | 7_Enh |  | H3K4me1_Enh |  |  |  |  |
| E090 | Muscle | MUS.LEG.FET | Fetal Muscle Leg | 7_Enh | 12_TxEnhW | H3K4me1_Enh |  | H3K27ac_Enh |  |  |
| E083 | Heart | HRT.FET | Fetal Heart |  |  | H3K4me1_Enh |  |  |  |  |
| E104 | Heart | HRT.ATR.R | Right Atrium |  |  | H3K4me1_Enh |  |  |  |  |
| E095 | Heart | HRT.VENT.L | Left Ventricle |  |  | H3K4me1_Enh |  |  |  |  |
| E105 | Heart | HRT.VNT.R | Right Ventricle |  |  |  |  |  |  |  |
| E065 | Heart | VAS.AOR | Aorta |  |  |  |  |  |  |  |
| E078 | Sm. Muscle | GI.DUO.SM.MUS | Duodenum Smooth Muscle |  |  | H3K4me1_Enh |  |  |  |  |
| E076 | Sm. Muscle | GI.CLN.SM.MUS | Colon Smooth Muscle |  |  | H3K4me1_Enh |  |  |  |  |
| E103 | Sm. Muscle | GI.RECT.SM.MUS | Rectal Smooth Muscle |  |  |  | H3K4me3_Pro |  |  |  |
| E111 | Sm. Muscle | GI.STMC.MUS | Stomach Smooth Muscle |  |  | H3K4me1_Enh |  |  |  |  |
| E092 | Digestive | GI.STMC.FET | Fetal Stomach |  |  | H3K4me1_Enh |  |  |  |  |
| E085 | Digestive | GI.S.INT.FET | Fetal Intestine Small |  |  |  |  |  |  |  |
| E084 | Digestive | GI.L.INT.FET | Fetal Intestine Large |  |  | H3K4me1_Enh |  |  |  |  |
| E109 | Digestive | GI.S.INT | Small Intestine | 7_Enh |  | H3K4me1_Enh |  |  |  |  |
| E106 | Digestive | GI.CLN.SIG | Sigmoid Colon |  | 12_TxEnhW | H3K4me1_Enh | H3K4me3_Pro | H3K27ac_Enh |  |  |
| E075 | Digestive | GI.CLN.MUC | Colonic Mucosa |  |  | H3K4me1_Enh |  | H3K27ac_Enh |  |  |
| E101 | Digestive | GI.RECT.MUC.29 | Rectal Mucosa Donor 29 |  |  | H3K4me1_Enh |  | H3K27ac_Enh | H3K9ac_Pro |  |
| E102 | Digestive | GI.RECT.MUC.31 | Rectal Mucosa Donor 31 |  |  | H3K4me1_Enh |  |  |  |  |
| E110 | Digestive | GI.STMC.MUC | Stomach Mucosa |  |  | H3K4me1_Enh |  |  | H3K9ac_Pro |  |
| E077 | Digestive | GI.DUO.MUC | Duodenum Mucosa | 7_Enh |  | H3K4me1_Enh | H3K4me3_Pro |  |  |  |
| E079 | Digestive | GI.ESO | Esophagus |  |  | H3K4me1_Enh |  |  |  |  |
| E094 | Digestive | GI.STMC.GAST | Gastric |  |  |  |  |  |  |  |
| E099 | Other | PLCNT.AMN | Placenta Amnion |  |  | H3K4me1_Enh |  |  |  |  |
| E086 | Other | KID.FET | Fetal Kidney |  |  |  |  |  |  |  |
| E088 | Other | LNG.FET | Fetal Lung |  |  |  |  |  | H3K9ac_Pro |  |
| E097 | Other | OVRY | Ovary |  |  |  |  |  |  |  |
| E087 | Other | PANC.ISLT | Pancreatic Islets |  |  |  |  |  |  |  |
| E080 | Other | ADRL.GLND.FET | Fetal Adrenal Gland |  |  |  |  |  |  |  |
| E091 | Other | PLCNT.FET | Placenta |  |  |  |  |  |  |  |
| E066 | Other | LIV.ADLT | Liver |  |  | H3K4me1_Enh |  | H3K27ac_Enh |  |  |
| E098 | Other | PANC | Pancreas |  |  |  |  |  |  |  |
| E096 | Other | LNG | Lung |  |  | H3K4me1_Enh |  |  |  |  |
| E113 | Other | SPLN | Spleen | 7_Enh | 12_TxEnhW | H3K4me1_Enh |  | H3K27ac_Enh |  |  |
| E114 | ENCODE2012 | LNG.A549.ETOH002.CNCR | A549 EtOH 0.02pct Lung Carcinoma Cell Line |  |  |  |  |  |  |  |
| E115 | ENCODE2012 | BLD.DND41.CNCR | Dnd41 TCell Leukemia Cell Line | 7_Enh | 9_TxReg | H3K4me1_Enh |  | H3K27ac_Enh | H3K9ac_Pro |  |
| E116 | ENCODE2012 | BLD.GM12878 | GM12878 Lymphoblastoid Cells | 7_Enh | 9_TxReg | H3K4me1_Enh | H3K4me3_Pro | H3K27ac_Enh | H3K9ac_Pro |  |
| E117 | ENCODE2012 | CRVX.HELAS3.CNCR | HeLa-S3 Cervical Carcinoma Cell Line |  |  |  |  |  |  |  |
| E118 | ENCODE2012 | LIV.HEPG2.CNCR | HepG2 Hepatocellular Carcinoma Cell Line |  |  |  |  |  |  |  |
| E119 | ENCODE2012 | BRST.HMEC | HMEC Mammary Epithelial Primary Cells |  |  |  |  |  |  |  |
| E120 | ENCODE2012 | MUS.HSMM | HSMM Skeletal Muscle Myoblasts Cells |  |  | H3K4me1_Enh |  |  |  |  |
| E121 | ENCODE2012 | MUS.HSMMT | HSMM cell derived Skeletal Muscle Myotubes Cells |  |  | H3K4me1_Enh |  |  |  |  |
| E122 | ENCODE2012 | VAS.HUVEC | HUVEC Umbilical Vein Endothelial Primary Cells |  |  |  |  |  |  |  |
| E123 | ENCODE2012 | BLD.K562.CNCR | K562 Leukemia Cells |  | 12_TxEnhW | H3K4me1_Enh |  |  |  |  |
| E124 | ENCODE2012 | BLD.CD14.MONO | Monocytes-CD14+ RO01746 Primary Cells | 2_TssAFlnk | 9_TxReg | H3K4me1_Enh | H3K4me3_Pro | H3K27ac_Enh | H3K9ac_Pro |  |
| E125 | ENCODE2012 | BRN.NHA | NH-A Astrocytes Primary Cells |  |  | H3K4me1_Enh |  |  |  |  |
| E126 | ENCODE2012 | SKIN.NHDFAD | NHDF-Ad Adult Dermal Fibroblast Primary Cells |  |  |  |  |  |  |  |
| E127 | ENCODE2012 | SKIN.NHEK | NHEK-Epidermal Keratinocyte Primary Cells |  |  |  |  |  |  |  |
| E128 | ENCODE2012 | LNG.NHLF | NHLF Lung Fibroblast Primary Cells |  |  |  |  |  |  |  |
| E129 | ENCODE2012 | BONE.OSTEO | Osteoblast Primary Cells |  |  |  |  |  |  |  |

**Figure S3. Regional association plot for the association with SLE risk independently from *GRB2* SNP rs36023980.** Log10(p values) of the association test conditioning on SNP rs36023980 at *GRB2* locus are plotted for **A.** *GRB2* locus SNP +/-1Mb and **B.** zooming on the independently associated SNP (rs9891273, P=4.99x10^-5^).

**A**
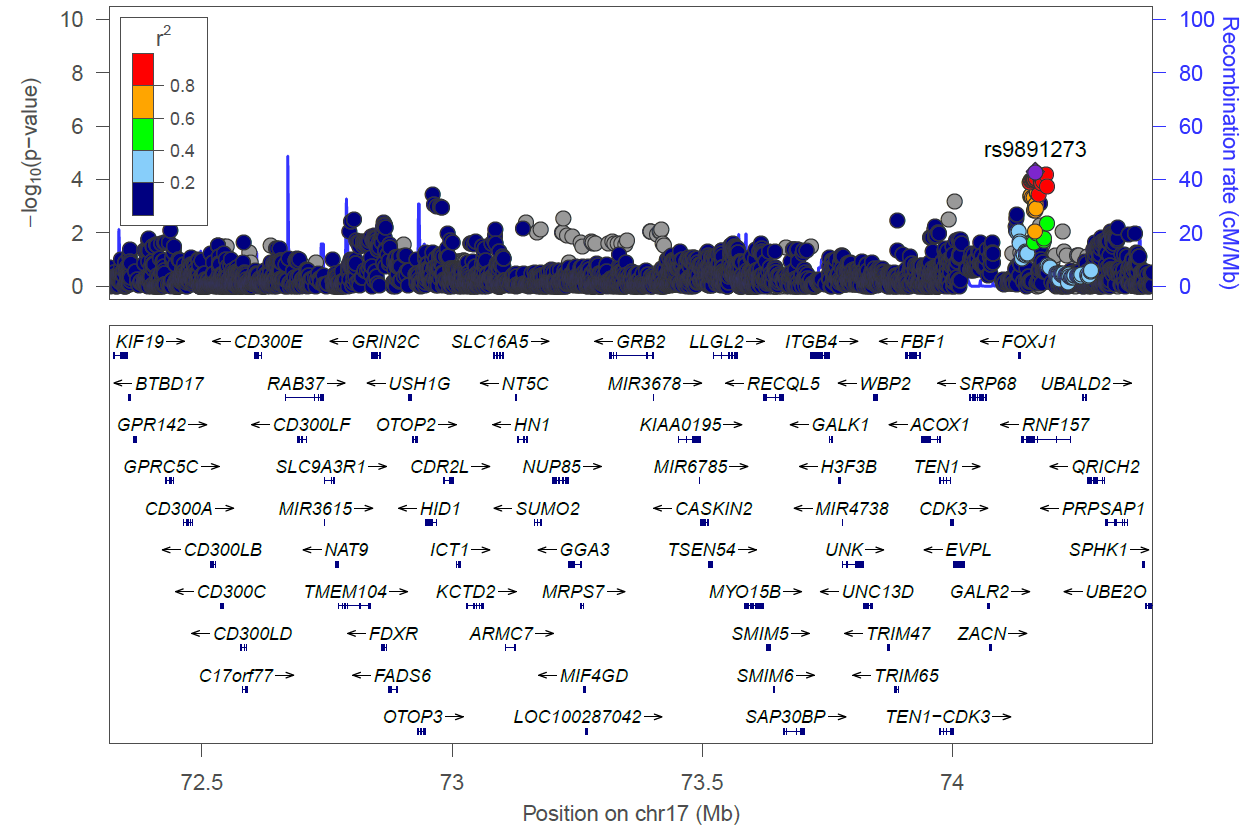


**B**
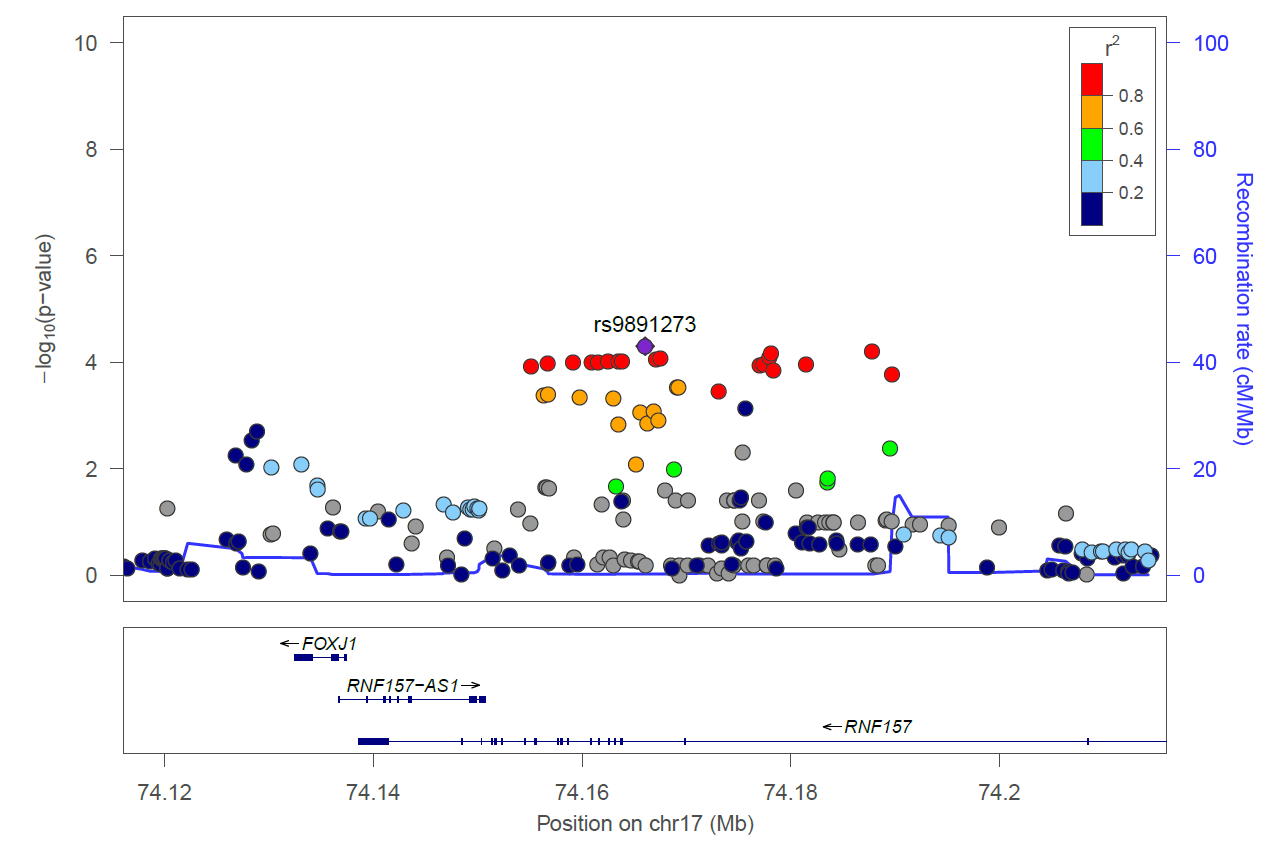


**Figure S4. *GRB2* gene expression during human B-cell differentiation.** Gene expression data for *GRB2* was obtained from B cell subpopulations during different differentiation stages using Affymetrix Human Genome U219 array as described in Kulis et al (*Whole-genome fingerprint of the DNA methylome during human B cell differentiation*. Nat Genet ’15). The dataset was generated by the Blueprint project with reference *EGAS00001001197*. Pre-B-I: bone marroy Pre-B-I committed B cell progenitor; Pre-B-2: bone marroy Pre-B-II committed B cell progenitor; NaiveB: resting naïve B cells from whole blood; GCB: germinal center B cells; memB: memory B cells from peripheral blood; PC: short-lived tonsillar plasma cells. *GRB2* expression increases in differentiated B cell subpopulations, reaching its highest levels on class-switched memory B cells.


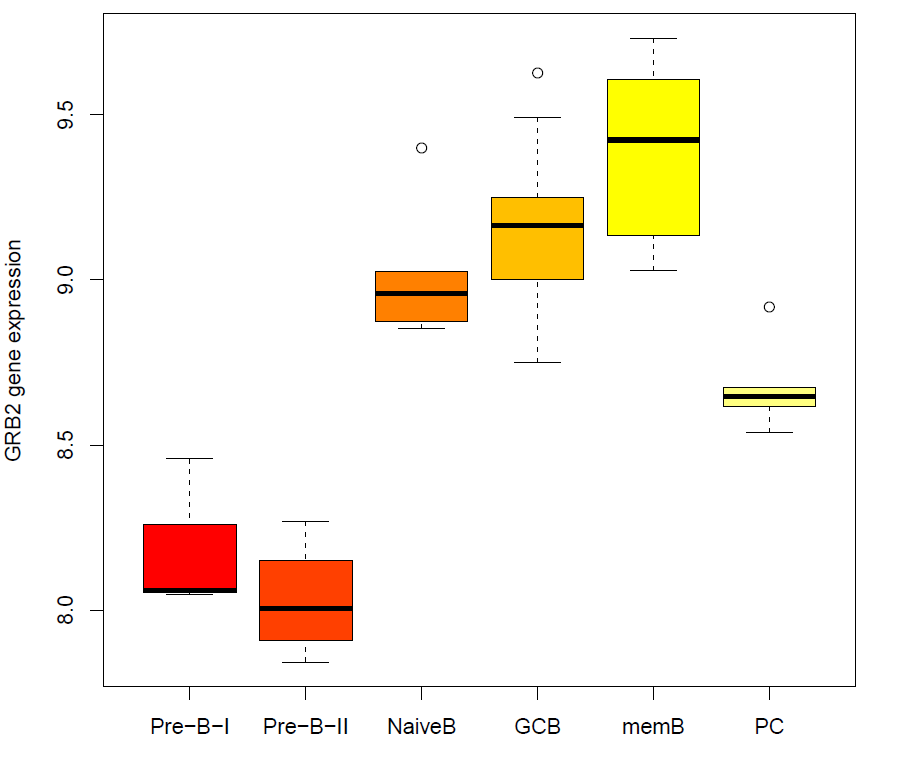

Supplement: Supplementary file 1 — Table S1. Epidemiological features from the Spain GWAS cohort. Figure S1. Principal component analysis of the Spain GWAS cohort. Figure S2. Quantile-quantile(Q-Q) plots of observed and expected -log10(p values) of association between SNP genotype and SLE risk. Table S2. Epigenetic regulatory data associated with GRB2 risk locus. Figure S3. Regional association plot for the association with SLE risk independent of GRB2 SNP rs36023980. Figure S4. GRB2 gene expression during human B cell differentiation. (DOCX 627 kb) [file 13075_2018_1604_MOESM1_ESM.docx]
